# Surgical Oncology Heroes and Legends: David P. Winchester, MD, as Interviewed by Stephen F. Sener, MD

**DOI:** 10.1245/s10434-025-19040-8

**Published:** 2026-01-12

**Authors:** David P. Winchester, Stephen F. Sener

**Affiliations:** 1 American College of Surgeons, Chicago, IL USA; 2 Keck School of Medicine of USC, Los Angeles, CA USA

The Surgical Oncology Heroes and Legends series is a video program in which leaders of the field are interviewed by colleagues about their many contributions. In succinct 30-minute conversations, these luminaries discuss with other leaders their experiences and insights about surgical oncology.

This episode presents an interview of David P. Winchester, MD by Stephen Sener, MD; watch the video (tem link: https://vimeo.com/user22936519/review/1060576238/ab0316f995).

*About David P. Winchester, MD*

Dr. David P. Winchester graduated from Northwestern University and received his general surgical training at Northwestern (Fig. 1). He completed his surgical oncology fellowship at M.D. Anderson Cancer Center. He has served as Clinical Professor of Surgery at the University of Chicago, Pritzker School of Medicine; Professor of Surgery, Northwestern University; and the immediate past Chairman of the Department of Surgery of NorthShore University HealthSystem (formerly Evanston Northwestern Healthcare). Dr. Winchester’s pivotal leadership roles have included the Medical Director of Cancer Programs at the American College of Surgeons (ACS) and Principal Investigator of the National Cancer Database (NCDB) with a focus on tracking outcomes in patients with cancer and promoting continuous quality improvement of cancer care. Dr. Winchester has authored more than 250 peer-reviewed journal articles, served on 10 editorial boards, and edited/co-edited 5 books. He is a Past-President of the Society of Surgical Oncology and past Executive Director of the American Joint Committee on Cancer. He is also the past Chairman of the National Accreditation Program of Breast Centers (NAPBC) for the ACS. He is the recipient of the first annual Society of Surgical Oncology Distinguished Service Award.

**Fig. 1:**
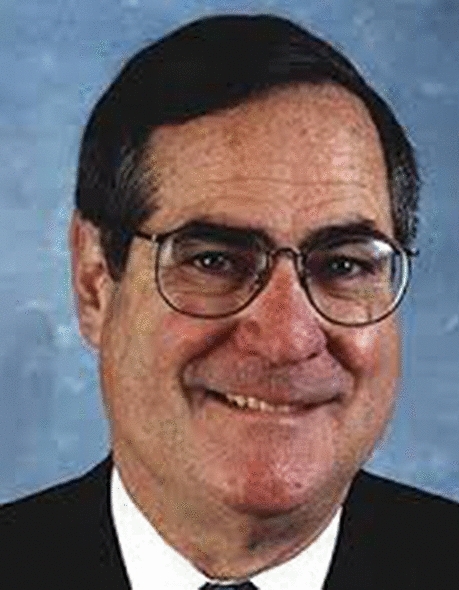
David P. Winchester, MD

*About Stephen F. Sener, MD*

Dr. Sener graduated from Northwestern University Medical School, followed by completion of the general surgery residency training program at Northwestern University. This training included 6 months at the National Cancer Institute in Dr. Steven Rosenberg’s lab and 1 year as the American Cancer Society Clinical Fellow with Dr. Edward Scanlon at Evanston Hospital. After finishing his general surgery residency training, Dr. Sener spent 2 years as a Surgical Oncology Fellow at Memorial Sloan-Kettering Cancer Center. Following this, in 1984, Dr. Sener returned to Northwestern University and Evanston Northwestern Healthcare (ENH) (now Endeavor Health). While there, Dr. Sener was promoted to Professor of Surgery at Northwestern University Feinberg School of Medicine and to Vice-Chairman of the Department of Surgery at ENH. He served as Associate Director for the Residency Training Program in Surgery at Northwestern from 2000 to 2002 and in 2007. In September 2009, Dr. Sener was recruited to the University of Southern California (USC) Department of Surgery to become chief of a newly created Division of Surgical Oncology. He was the Program Director for the USC-Hoag Breast Surgical Oncology Fellowship from 2009 to 2020 and has been Associate Program Director for the USC General Surgery Residency Training Program from 2012 to present. In April 2015, he was appointed Chief of Surgery and Medical Director for Perioperative Services at Los Angeles General Medical Center (formerly LAC+USC Hospital). Dr. Sener has been a long-term volunteer for the American Cancer Society (ACS) and has served as the ACS representative to the American Joint Committee on Cancer, the Commission on Cancer, and the Society of Surgical Oncology. He was the National President of the ACS in 2004–2005. One of Dr. Sener’s most gratifying projects of his professional career was the establishment, on behalf of the ACS and the Chinese government, of the One Million Women Program for breast cancer screening mammography in China in 2005.

In addition to having served on the Editorial Boards of *Cancer Practice*, *Journal of Surgical Oncology*, *American Journal of Surgery*, and the *Journal of Clinical Oncology*, Dr. Sener served as Editor-in-Chief of the *Journal of Surgical Oncology* from 2013 to 2024.

## Brief Bibliographies: Top Articles by Dr. Winchester and Dr. Sener

Dr. Winchester and Dr. Sener were invited to identify the five most important articles of which they are the sole author or a co-author. They provided the following lists:

### Pivotal Articles Authored or Co-Authored by Dr. David P. Winchester

1. Amin MB, Greene FL, Edge SB, et al. The Eighth Edition AJCC Cancer Staging Manual: Continuing to build a bridge from a population-based to a more “personalized” approach to cancer staging. *CA Cancer J Clin*. 2017;67:93–99
2. Bilimoria KY, Stewart AK, Winchester DP, et al. The National Cancer Data Base: a powerful initiative to improve cancer care in the United States. *Ann Surg Oncol*. 2008;15:683–90.
3. Boffa DJ, Rosen JE, Mallin K, et al. Using the national cancer database for outcomes research: a review. *JAMA Oncol*. 2017;3(12):1722–8.
4. Giuliano AE, Connolly JL, Edge SB, et al. Breast cancer—Major changes in the American Joint Committee on Cancer eighth edition cancer staging manual. *CA Cancer J Clin*. 2017; 67:290-303
5. Sener SF, Fremgen A, Menck HR, Winchester DP. Pancreatic cancer: a report of treatment and survival trends for 100,313 patients diagnosed from 1985–1995, using the National Cancer Database. *J Am Coll Surg*. 1999;189(1):1–7.

### Pivotal Articles Authored or Co-Authored by Dr. Stephen Sener

1. Winchester DJ, Sener SF, Winchester DP, Brenin D, Pearlman RM, Stull MA, Moulthrop JM, Goldschmidt RA, Motykie G, Martz C, Rabbitt S. Sentinel lymphadenectomy for breast cancer: experience with 180 consecutive patients: efficacy of filtered technetium 99m sulphur colloid with overnight migration time. *J Am Coll Surg*. 1999;188:597–603.
2. Sener SF, Winchester DJ, Winchester DP, Kurek R, Motykie G, Martz CH, Rabbitt S. Spectrum of mammographically-detected breast cancer. *Am Surg*. 1999;65(8):731–6.
3. Sener SF, Winchester DJ, Martz CH, Feldman JL, Cavanaugh JA, Winchester DP, Weigel B, Bonnefoi K, Kirby K, Morehead C. Lymphedema following sentinel lymphadenectomy for breast carcinoma. *Cancer*. 2001;92:748–52.
4. Saslow D, Boetes C, Burke W, et al. American Cancer Society Guidelines for breast screening with MRI as an adjunct to mammography. *CA Cancer J Clin*. 2007;57(2):75–89.
5. Hand R, Fremgen A, Chmiel JS, et al. Staging procedures, clinical management, and survival outcome for ovarian carcinoma. *JAMA*. 1993;269(9):1119–22.

